# 738. Outcomes of Ceftriaxone Versus Antistaphylococcal Penicillins or Cefazolin for Definitive Therapy of Methicillin-susceptible *Staphylococcus aureus* Bacteremia

**DOI:** 10.1093/ofid/ofac492.029

**Published:** 2022-12-15

**Authors:** Ryan B Khodadadi, Zachary A Yetmar, John R Go, Supavit Chesdachai, Samrah Razi, Omar M Abu Saleh

**Affiliations:** Mayo Clinic, Rochester, Minnesota; Mayo Clinic, Rochester, Minnesota; Division of Public Health, Infectious Diseases, and Occupational Medicine, Department of Medicine, Mayo Clinic, Rochester, Minnesota; Mayo Clinic, Rochester, Minnesota; Mayo Clinic, Rochester, Minnesota; Division of Public Health, Infectious Diseases, and Occupational Medicine, Department of Medicine, Mayo Clinic, Rochester, Minnesota

## Abstract

**Background:**

Methicillin-susceptible *Staphylococcus aureus* (MSSA) bacteremia requires weeks of IV antimicrobial therapy for treatment with antistaphylococcal penicillins (ASP) and first-generation cephalosporins being mainstays of therapy. While effective, use of these agents in the outpatient setting requires frequent dosing along with close toxicity monitoring through the duration of therapy. Ceftriaxone is an appealing alternative given daily dosing and tolerability, but efficacy in this setting remains uncertain. We sought to review outcomes of patients who received ceftriaxone versus an ASP and first-generation cephalosporins for definitive therapy of MSSA bacteremia.

**Methods:**

Following IRB approval, we reviewed medical records of a retrospective adult cohort diagnosed with MSSA bacteremia treated with ceftriaxone, cefazolin, or an ASP between January 2018 and December 2019 at Mayo Clinic campuses. Treatment was defined as the antibiotic used in the outpatient setting. The primary outcome was 90-day treatment failure, defined as a composite of mortality and microbiologic relapse. Secondary outcomes were 30-day treatment failure and individual components of treatment failure. Multivariable Cox regression analysis was performed to analyze for an association between ceftriaxone use and the primary outcome after adjusting for factors that may also impact outcomes. Kaplan-Meier curves were compared utilizing a log-rank test for survival between groups.

**Results:**

A total of 223 patients with the accompanying characteristics met study criteria (Table 1). Thirty-seven received ceftriaxone (16.5%) and 186 (83.4%) were treated with cefazolin or an ASP. Ninety and 30-day treatment failure were higher in those treated with ceftriaxone (27%, 8.1%) compared to cefazolin or an ASP (8.6%, 3.8%). Multivariable analysis for risk of 90-day treatment failure revealed a hazard ratio of 2.91 for ceftriaxone (Table 2) and a significant difference in event-free survival for those treated with ceftriaxone compared to cefazolin or an ASP (Figure 1).

**Conclusion:**

Patients receiving definitive therapy with ceftriaxone for MSSA bacteremia experienced a higher rate of 90 and 30-day treatment failure and a lower rate of event-free survival as compared to those treated with cefazolin or an ASP.

**Disclosures:**

**All Authors**: No reported disclosures.

**Table 1:**
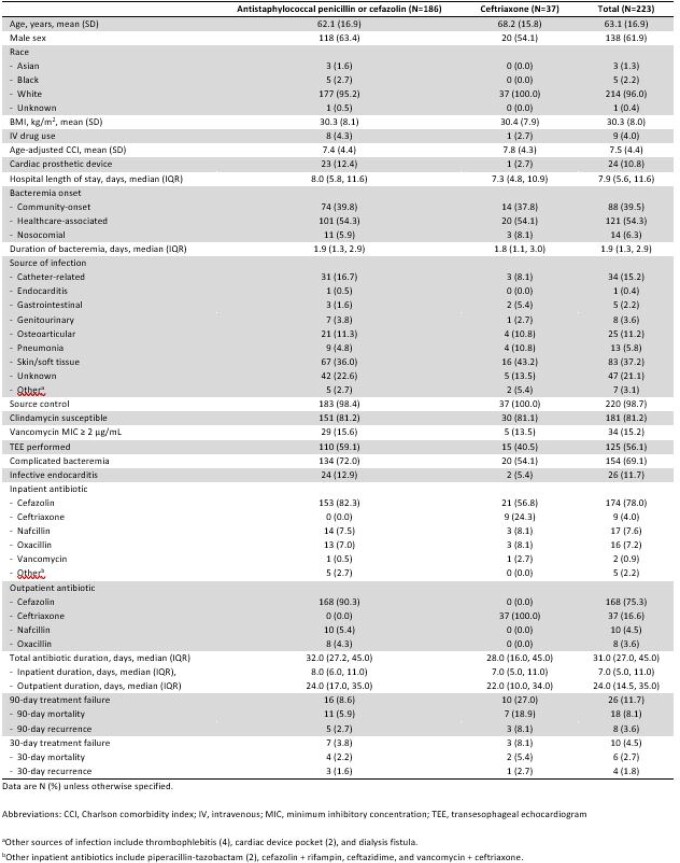
Characteristics of 223 patients with methicillin-susceptible Staphylococcus aureus bacteremia.

**Table 2:**
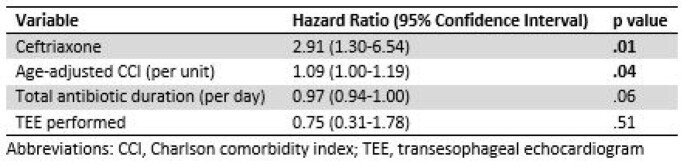
Multivariable Cox proportional hazards model for risk of 90-day treatment failure.

**Figure 1:**
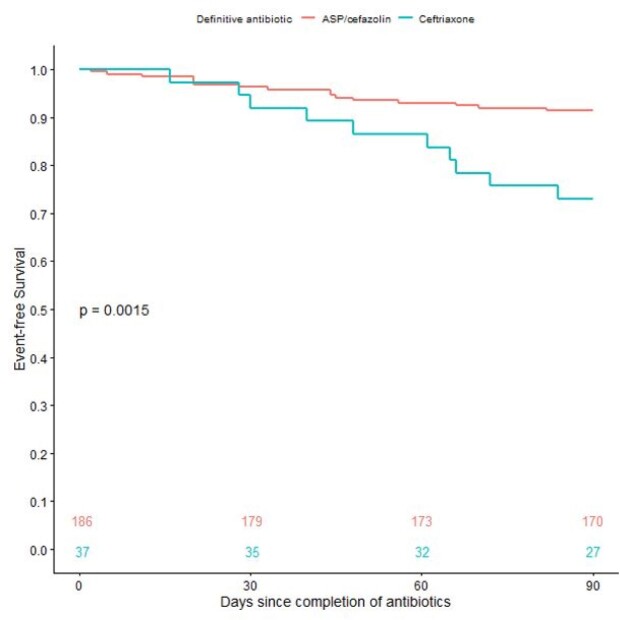
Kaplan-Meier curve comparing 90-day treatment failure by definitive antibiotic between antistaphylococcal penicillins or cefazolin and ceftriaxone. The p-value is calculated via the log-rank test.

